# Efficacy and Tolerability of a Microneedling Device for Treating Wrinkles on the Neck

**DOI:** 10.1093/asj/sjac085

**Published:** 2022-04-09

**Authors:** Mona Alqam, Christine E Wamsley, Thomas Hitchcock, Brian C Jones, Yucel Akgul, Jeffrey M Kenkel

**Affiliations:** Crown Laboratories, Dallas, TX, USA; Department of Plastic Surgery, UT Southwestern Medical Center, Dallas, TX, USA; Crown Laboratories, Dallas, TX, USA; Crown Laboratories, Dallas, TX, USA; Department of Plastic Surgery, UT Southwestern Medical Center, Dallas, TX, USA; Department of Plastic Surgery, UT Southwestern Medical Center, Dallas, TX, USA

## Abstract

**Background:**

A microneedling pen has been cleared by the US FDA and is indicated for improving the appearance of facial acne scars in adults.

**Objectives:**

The aim of this study was to assess the effectiveness of this microneedling pen for treating wrinkles. This paper focuses on the results on the neck, an area of recent importance with video meetings placing physical stress on the neck area, leading to wrinkles.

**Methods:**

Healthy adult men and women were enrolled (N = 35). Subjects received 4 monthly microneedling procedures at depths of up to 2.5 mm. Wrinkle assessments were performed by 2 trained blinded raters by comparing images of each subject at baseline and at 90 days postprocedure. The 2 raters were unblinded for the Clinician’s Global Aesthetic Improvement Scale assessment. Subjects completed the Subject’s Global Aesthetic Improvement Scale and a questionnaire regarding satisfaction with the treated areas of the face and neck at 30 and 90 days posttreatment.

**Results:**

The study was completed by 32 subjects. Wrinkle assessments demonstrated significant improvement in the neck areas (*P* < 0.001). Both Global Aesthetic Improvement Scales showed significant improvements at 90 days posttreatment (*P* < 0.001). Most subjects reported some level of improvement in their appearance at 30 days (73.3%) and 90 days (68.8%) posttreatment. The satisfaction questionnaire showed high levels of improvement in wrinkles (93.8%), satisfaction with the results (87.5%), and would recommend microneedling to friends and family members (80.6%).

**Conclusions:**

Microneedling is a viable, minimally invasive option for treating wrin kles of the neck.

**Level of Evidence: 4:**

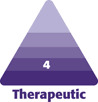

See the Commentary on this article here.

During the past decade, microneedling has gained popularity as a safe, effective, and affordable aesthetic procedure.^1^ Like many other rejuvenation techniques, microneedling is a method of mechanically inducing skin remodeling.^2,3^ It is a minimally invasive procedure consisting of controlled, superficial puncturing of the skin with fine needles^4^ which stimulates the normal wound healing process whereby an initial inflammatory reaction is followed by proliferation of the extracellular matrix and remodeling of new dermal tissues.^5^

Physiological changes associated with microneedling include upregulation of genes associated with tissue remodeling and wound healing, epithelial proliferation and differentiation, immune cell recruitment, and downregulation of proinflammatory cytokines.^6^ Microneedling significantly increases baseline collagen types I, III, and VII, newly synthesized collagen, and tropoelastin.^7^ As a result, microneedling therapy is used to improve the appearance of facial scars,^8,9^ stretch marks,^10,11^ photoaged skin,^7^ and dyschromia conditions. When used as a drug delivery system, microneedling has be used to treat alopecia, pigmentary disorders, and actinic keratoses.^12^

An automated, nonsurgical microneedling pen was the first to be cleared by the US FDA as a microneedling device (SkinPen Microneedling System; Crown Aesthetics, Dallas, TX) and was originally cleared with the indication as a procedure for improving the appearance of facial acne scars in adults aged 22 years or older; however, recent evidence supports the use of microneedling for treating skin rhytides.^13-15^ Based on these promising results, the following study was performed to assess the effectiveness of the microneedling pen for treating wrinkles on the face and neck. This paper is a subjective endpoints companion to the previously published objective endpoints paper, where noninvasive measurements and biopsy data of the face showed changes in skin architecture and collagen/elastin gene expression, suggesting skin rejuvenation, with new extracellular matrix production and muscle formation.^16^

## METHODS

### Study Subjects

Eligible subjects were healthy men and women, aged 35 to 65 years old, seeking treatment to improve the appearance of wrinkles on the face and neck. Written consent was provided, by which the patients agreed to the use and analysis of their data. Each subject expressed their willingness to comply with all study requirements and refrain from prohibited procedures including soft tissue fillers, resurfacing therapies, botulinum toxins, injectable fillers, microdermabrasion, laser and light procedures, skin tightening, or laser facial hair removal for the duration of the study. Waxing and threading were allowed. Women of childbearing potential were required to provide a negative urine pregnancy test at the baseline and 3-month posttreatment visits and agreed to use an acceptable method of birth control during the study.

Subjects were excluded from participation if they had known allergies to skincare products or topical lidocaine; a systemic or local disease or condition or medication affecting wound healing or any uncontrolled systemic disease; severe solar elastosis; recent trauma or scarring other than acne scars on the planned treatment area; severe or clinically significant acne on the planned treatment area, defined as >5 active inflammatory acne lesions including acne conglobate, nodules, or cysts in a planned treatment area; a history of hypertrophic or keloid scars; cancerous or precancerous lesions in the planned treatment areas or a history of skin cancer; inability to understand instructions or provide informed consent; history of chronic drug or alcohol abuse; undergoing concurrent therapy that might place the subject at risk or jeopardize study objectives; current smoker or smoked in the last 5 years; had undergone the following cosmetic treatments (time frame) in the planned treatment area: microdermabrasion or glycolic acid treatment (1 month), skin tightening (1 year), injectable filler including hyaluronic acid (12 months), calcium hydroxylapatite (12 months), poly-l-lactic (24 months) or permanent fillers (ever); neurotoxins (3 months); ablative laser resurfacing, nonablative rejuvenative laser or light treatment (6 months); surgical dermabrasion, deep facial peels, chemical peels, or dermabrasion of the face or neck (4 weeks); isotretinoin or other systemic retinoids (6 months), topical retinoids (2 weeks) or prescription-strength skin hydroquinone, α-hydroxy acid, β-hydroxy acid, and polyhydroxy acids, 4-hydroxyanisole alone or in combination with tretinoin (4 months). Other exclusion criteria were: nursing, pregnant, or planning to become pregnant; immune deficiency disorders or immunosuppressive medications; recently started hormone replacement therapies (<3 months) or plan on changing the dose of their therapy during the study; planned surgeries, overnight hospitalization, or invasive medical procedures during the study; and participation in any other study involving the use of investigational devices or drugs (4 weeks).

### Study Device

The microneedling handpiece is used with sterile, individually packaged, disposable needle cartridges. The pen and needle cartridge interface with a nonsterile, disposable sheath to prevent microneedling pen contamination (SkinPen Precision System). The 14 solid (0.25-mm) needles operate at a speed of 6300 to 7700 rpm with a maximum cartridge needle extension <2.5 mm.

### Study Procedures

During a 2-week baseline period prior to study onset (Visit 1), subject eligibility was confirmed, overall health and wrinkle severity assessments were performed, and female subjects completed a pregnancy test. Subjects were instructed not to use topical medications or retinoids for the 2 weeks prior to the first treatment and to maintain their current skincare routine with regular brands of color cosmetics and makeup remover and to refrain from the use of any antiaging and acne products or devices.

Trained aestheticians treated the wrinkles of the neck skin of each subject with the microneedling pen according to the manufacturer’s instructions^17^ at depths up to 2.5 mm at Visits 2 to 5 on Days 1, 30, 60, and 90. All treatment procedures were performed according to the manufacturer’s instruction manual.

Makeup was removed at least 30 minutes before each clinic visit with the provided facewash. Subjects were encouraged to avoid extended periods of sun exposure and any use of tanning beds for the duration of the study. Concomitant medications and health assessments were recorded during Visits 2 to 5 on Days 1, 30, 60, and 90. Pregnancy testing was repeated at Visit 7 at 90 days postprocedure. Subjects were provided with daily diaries at Visits 2 through 5 on Days 1, 30, 60, and 90. The completion of a daily diary was reviewed for safety and compliance and subjects received a new diary. Subjects were acclimated to ambient temperature and humidity conditions for 15 minutes before any study-related procedures were performed.

### Imaging Procedures

Before performing the imaging procedures, study personnel ensured the neck was free of makeup and jewelry was removed from the treatment area. Subjects were provided with a black or gray matte headband to keep hair away from the neck and a black or gray matte cloth was draped over subjects’ clothing. Digital images of the neck of each subject were obtained prior to treatment at visit Days 1, 30, 60, 90, and 30 and 90 days posttreatment (Nikon D710; Nikon Inc., Melville, NY).

For digital imaging, subjects were instructed to adopt neutral, nonsmiling expressions. Subjects were carefully positioned facing the camera for a center view and 45° right and left side views. The randomization of images was completed utilizing an online research randomizer tool (www.randomizer.org, Social Psychology Network, New York, NY).

### Imaging Assessments

Two trained raters assessed blinded randomized images of subjects prior to treatment (Day 1) and 90 days posttreatment. After completing wrinkle assessment, the 2 raters were unblinded to pretreatment images for the Clinician’s Global Aesthetic Improvement Scale (CGAIS).

### Subject Self-assessments

Each subject completed a sponsor-provided self-assessment questionnaire and the Subject’s Global Aesthetic Improvement Scale (SGAIS) at the 30- and 90-day posttreatment visits. The Patient Satisfaction Questionnaire included questions regarding improvement of fine lines and wrinkles, satisfaction with the treatment, and willingness to recommend the treatment to friends and family members. The survey was done on a paper form and was partially anonymous; it was anonymous to the sponsors (employees at Crown Laboratories, Dallas, TX) but not anonymous to the principal investigators and subinvestigators conducting the study. The investigators distributed the survey.

### Safety

At each study visit, subjects were queried about potential adverse events by means of open-ended questions, and the treatment area was examined. The use of digital imaging was encouraged to document any adverse events.

### Study Endpoints

Clinical outcome and safety endpoints were based on clinic assessments and evaluation of pre- and posttreatment digital images, including the neck. Primary efficacy endpoints included assessment of wrinkle severity on a modified Lemperle Wrinkle Assessment Scale^18^ (Table 1), and CGAIS scores at baseline and 30 and 90 days posttreatment.

**Table 1.** Lemperle Wrinkle Assessment Scale

Grade	Severity
0	No wrinkles
1	Just perceptible wrinkles
2	Shallow wrinkles
3	Moderately deep wrinkles
4	Deep wrinkle, well-defined edges
5	Very deep wrinkles, redundant folds

SCAIS scores and the Patient Satisfaction Questionnaire were secondary efficacy endpoints. Safety was assessed by reported adverse events throughout the course of the study.

### Ethics

The protocol, the informed consent form, and other study-related documents were approved by the University of Texas Southwestern Medical Center IRB (Dallas, TX) according to 21 Code of Federal Regulations 50.25 requirements. The study was conducted between January 2019 and January 2020. Each enrolled subject provided a signed photography release. The study was conducted in accordance with the Declaration of Helsinki,^19^ and is registered with ClinicalTrials.gov (identifier: NCT0380305).

### Statistical Analysis Population

The intention-to-treat population included all subjects who received a baseline and at least 1 treatment assessment and completed the study in accordance with the protocol. The descriptive statistical summary includes the number of observations (N), mean, median, standard deviation (SD), minimum (min), and maximum (max) of values at all applicable time points. The primary intention-to-treat analysis is based on the primary outcome of Lemperle gradings for the neck at 3 months posttreatment (study endpoint) relative to baseline (Day 1) evaluation based on the post hoc photographic ratings of 2 blinded evaluators. An individual study responder is defined as having attained a grading improvement of 1 or more grades as determined by both blinded evaluators. Overall study success (responder rate) is defined as 50% or more subjects being an individual responder.

The mean change was determined for all applicable parameters. Satisfaction questionnaire results were tabulated, and a binomial (sign) test was performed to determine if the proportion of favorable responses was equal to negative responses. All statistical tests were 2-sided with α = 0.05. No multiple testing corrections were considered.

## RESULTS

### Safety Evaluations

No unanticipated adverse events associated with the treatment were seen in the study.

### Subject Demographics

Among the enrolled subjects (N = 35), 32 completed the study and comprised the study population; 30 were females (93.75%) and 2 (6.25%) were males. At the time of enrollment, the mean [standard deviation] age of subjects was 56.3 [4.96] years (range, 44-65 years). Subjects had 2 follow-up visits 1 month and 3 months posttreatment with a ±3-day visit window. Two subjects were lost to follow up and 1 was withdrawn for noncompliance. The demographics and baseline characteristics of the study subjects are summarized in Table 2.

**Table 2.** Demographics and Baseline Characteristics

Variable	Statistics
Mean age, years [SD]	56.3 [5.0]
Median age, years (min, max)	56.5 (44, 85)
Gender, n (%)	
Female	30 (93.8)
Male	2 (6.3)
Race, n (%)	
White or Caucasian	28 (87.5)
Other	4 (12.5)
Ethnicity, n (%)	
Non-Hispanic/Latino	28 (87.5)
Hispanic/Latino	4 (12.5)
Fitzpatrick skin type, n (%)	
II	24 (75.0)
III	4 (12.5)
IV	4 (12.5)

SD, standard deviation.

Analysis of the photograph grading by blinded reviewers at 90 days posttreatment revealed a significant decrease (improvement) in baseline scores for wrinkling on the neck. The mean scores between the 2 blinded raters were used in the analysis. These are summarized in Table 3.

**Table 3.** Change in Baseline Neck Wrinkles

Area	Improved (%)	Worsened (%)	Mean [SD]	Mean change (%)	*P* value ^a^
Neck	50	0.0	–0.86 [0.66]	–25.9	<0.001

SD, standard deviation.

^a^Wilcoxon signed rank test.

**Figure 1.**
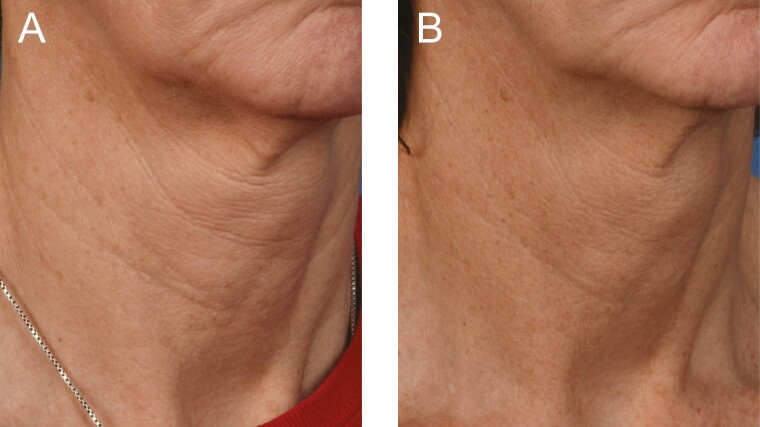
A 56-year-old female at (A) baseline and (B) 3 months after the last microneedling procedure.

**Figure 2.**
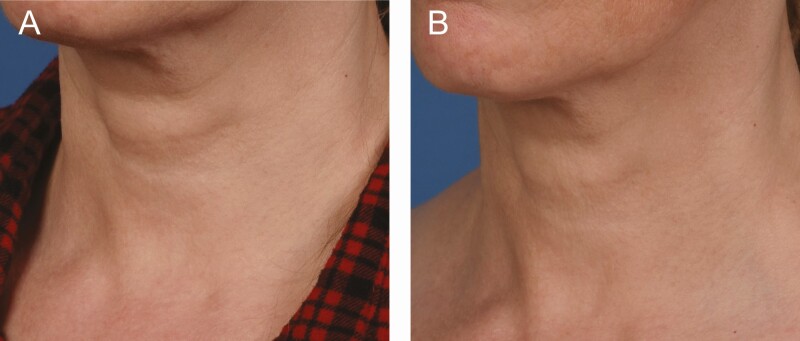
A 48-year-old female at (A) baseline and (B) 3 months after the last microneedling procedure.

**Figure 3.**
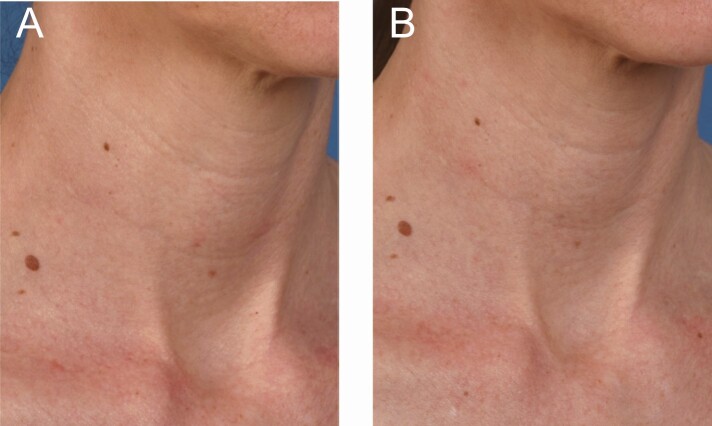
A 44-year-old female at (A) baseline and (B) 3 months after the last microneedling procedure.

**Figure 4.**
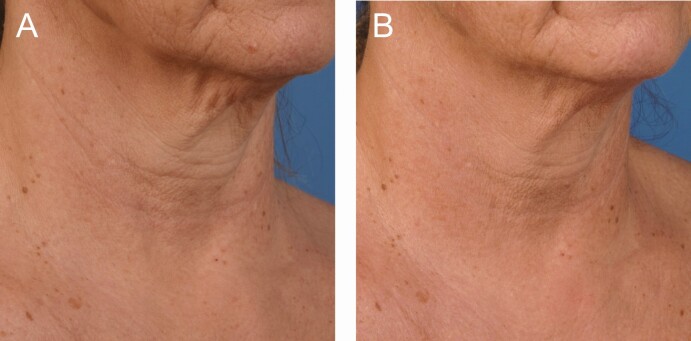
A 56-year-old female at (A) baseline and (B) 3 months after the last microneedling procedure.

As for assessment based on photographs by 2 reviewers after study completion, there was a significant improvement in the mean CGAIS at 3 months posttreatment (*P* < 0.001). These results are summarized in Table 4 and Figures 1 to 4.

**Table 4.** Descriptive Statistics for Global Aesthetic Improvement Assessment

Variable		Time posttreatment (months)	n	Mean [SD]	Median (min, max)	*P* value^a^	Very much improved, much improved, improved, n (%)	No change, n (%)	Worse, n (%)
Subject’s self-assessment for aesthetic improvement		1	30	2.90 [0.84]	3.0 (1.0, 4.0)	<0.001			
		3	32	3.16 [0.72]	3.0 (1.0, 4.0)	<0.001			
Clinician’s global aesthetic improvement assessment		3	32	3.06 [0.74]	3.0 (1.5, 4.5)	<0.001			
Frequency tabulation for global aesthetic improvement assessment	Subject’s self-assessment for aesthetic improvement	1	30				22 (73.3)	8 (26.7)	0
		3	32				22 (68.8)	10 (31.3)	0
	Clinician’s global aesthetic improvement assessment	3	32				26 (81.3)	4 (12.5)	2 (6.3)

SD, standard deviation.

^a^Calculated with the Wilcoxon signed rank test.

The SGAIS scores also showed significant improvements at 30 and 90 days posttreatment as evaluated by study subjects (*P* < 0.001 for both time points). Similarly, assessment by blinded reviewers demonstrated significant improvement in the mean CGAIS scores at 3 months posttreatment (*P* < 0.001) (Table 4). A majority of subjects reported some level of improvement in their appearance at 30 days (73.3%) and 90 days (68.8%) following final treatment, whereas most clinical assessments noted improvement after 90 days (81.3%) (Table 4).

The results of the Patient Satisfaction Questionnaire showed high levels of improvement in the appearance of wrinkles in the treated area (93.8%), satisfaction with the treatment procedure (87.5%), and would recommend this microneedling procedure to their friends and family members (80.6%) (Table 5). No unanticipated adverse events associated with the treatment were seen in the study.

**Table 5.** Patient Satisfaction Questionnaire Results

Question	Time posttreatment (months)	Favorable, n (%)	Unfavorable, n (%)	Neutral, n (%)	Yes, n (%)	No, n (%)	*P* value^a^
“Do you notice any improvement in how your wrinkles look in the treated area?”	1, n = 32	30 (93.8)	2 (6.3)	0			<0.001
	3, n = 32	23 (71.9)	9 (28.1)	0			0.020
“How would you characterize your satisfaction with the treatment?”	1, n = 32	28 (87.5)	3 (9.4)	1 (3.1)			<0.001
	3, n = 32	24 (75.0)	6 (18.8)	2 (6.3)			<0.001
“Would you recommend this treatment to your friends and family members?”	1, n = 31	25 (80.6)	6 (19.4)	0			<0.001
	3, n = 32	21 (65.6)	11 (34.4)	0			0.110
“Do you notice any improvement in how your wrinkles look in the treated area?”	1, n = 32				30 (93.8)	2 (6.3)	
	3, n = 32				23 (71.9)	9 (28.1)	
“Reduction in the number of wrinkles?”	1, n = 32				12 (37.5)	20 (62.5)	
	3, n = 32				12 (37.5)	20 (62.5)	
“Reduction in the size of wrinkles?”	1, n = 32				25 (78.1)	7 (21.9)	
	3, n = 32				17 (53.1)	15 (46.9)	
“Reduction in pore size?”	1, n = 32				11 (34.4)	21 (65.6)	
	3, n = 32				11 (34.4)	21 (65.6)	
“Smoother skin texture?”	1, n = 32				20 (62.5)	12 (37.5)	
	3, n = 32				15 (46.9)	17 (53.1)	
“More even skin tone/color?”	1, n = 32				13 (40.6)	19 (59.4)	
	3, n = 32				9 (28.1)	23 (71.9)	
“Would you recommend this treatment to your friends and family members?”	1, n = 31				25 (80.6)	6 (19.4)	
	3, n = 32				21 (65.6)	11 (34.4)	

^a^Binomial (sign) test.

## DISCUSSION

Microneedling is a means to induce localized dermal tissue remodeling to improve skin texture, scars, and wrinkles. The microneedling device used in the present study is cleared as a procedure for improving the appearance of facial acne scars in adult patients; however, it also has been shown here to be a highly effective means for improving the appearance of wrinkles on the neck. There were significant improvements in all measures of efficacy at 30 days following a series of 4 monthly microneedling procedures. The effects were durable, persisting for at least 90 days following the last treatment. Data from this study was submitted to the FDA for review of benefits of the device, which was subsequently cleared for the indication for the improvement of neck wrinkles.

Significant improvement in the appearance of wrinkling on the neck (78.1%) was observed. No subject showed a worsening of wrinkle appearance. The majority of subjects believed their overall appearance was improved after 30 days (73.3%) and 90 days (68.8%) posttreatment and none believed it had worsened.

Most changes were noted by subjects at 30 days posttreatment. At that time, most subjects noted improvement in the appearance of wrinkles in the treated area (93.8%), were satisfied with treatment (87.5%), and would recommend this treatment to their friends and family members (80.6%). These results decreased but remained significant at 90 days posttreatment.

Originally developed as a roller device for treating acne scars,^20^ microneedling has advanced to produce more sophisticated devices that deliver high operating speeds, significantly more microinjuries per cm^2^, and more accurate penetration depths—all of which greatly enhance their precision and clinical outcomes. Overall, these results add to the growing body of data that support the use of microneedling for skin rejuvenation and the expanding use of this versatile procedure for numerous clinical applications. Limitations to this study include a limited population size, lack of a longer follow-up period, and a bias for White, female subjects due to the difficulty finding higher Fitzpartrick skin types meeting inclusion criteria.

## CONCLUSIONS

Microneedling is a safe, viable, minimally invasive option for treating wrinkles of the neck. Significant improvements were noted as early as 30 days following 4 monthly treatments. Overall patient satisfaction was high. Microneedling may provide beneficial effects for aesthetic and medical dermal conditions other than on face and neck areas, but this requires further study.

## Supplementary Material

sjac085_suppl_Supplementary_Material
