# Supplementary material for: Efficacy and Tolerability of a Microneedling Device for Treating Wrinkles on the Neck
Source: Aesthet Surg J. 2022 Apr 9;42(10):1154–60. doi: 10.1093/asj/sjac085 (PMC9472661; doi:10.1093/asj/sjac085)
Supplement: sjac085_suppl_Supplementary_Material [file sjac085_suppl_supplementary_material.pdf]

Patient Satisfaction Questionnaire

PLEASE HAVE THE SUBJECT COMPLETE THIS ASSESSMENT WHILE REFERRING TO BASELINE PHOTOS.

COMPARE HOW YOUR FACE CURRENTLY LOOKS WITH YOUR PRE-TREATMENT PHOTOS

Please indicate what you think about how the treated areas of your fine lines and wrinkles look today. Look in the mirror at the treated area on both sides of your face and compare to the photos taken of both sides of your face prior to your study treatment.

1. Do you notice any improvement in how your fine lines and wrinkles look in the treated area?

   YES

☐ Reduction in the number of fine lines and wrinkles
☐ Reduction in the size of fine lines and wrinkles
☐ Reduction in pore size
☐ Clearer skin
☐ Smoother skin texture
☐ More even skin tone (color)

Other:

☐ NO

2. How would you characterize your satisfaction with the treatment?

☐ Extremely Satisfied
☐ Satisfied
☐ Slightly Satisfied
☐ Neither Satisfied or Dissatisfied
☐ Slightly Dissatisfied
☐ Dissatisfied
☐ Very Dissatisfied

3. Would you recommend this treatment to your friends and family members *(check one)*?

YES NO

Thank you for completing this questionnaire.

Subject Initials: _______________ Date: ______________________ (MM/DD/YYYY)
